# Three-terminal resistive switch based on metal/metal oxide redox reactions

**DOI:** 10.1038/s41598-017-06954-x

**Published:** 2017-08-07

**Authors:** Mantao Huang, Aik Jun Tan, Maxwell Mann, Uwe Bauer, Raoul Ouedraogo, Geoffrey S. D. Beach

**Affiliations:** 1 Department of Materials Science and Engineering, Massachusetts Institute of Technology, Cambridge, Massachusetts, 02139 USA; 2 MIT Lincoln Laboratory, Lexington, Massachusetts 02421-6426 USA

## Abstract

A solid-state three-terminal resistive switch based on gate-voltage-tunable reversible oxidation of a thin-film metallic channel is demonstrated. The switch is composed of a cobalt wire placed under a GdOx layer and a Au top electrode. The lateral resistance of the wire changes with the transition between cobalt and cobalt oxide controlled by a voltage applied to the top electrode. The kinetics of the oxidation and reduction process are examined through time- and temperature-dependent transport measurements. It is shown that that reversible voltage induced lateral resistance switching with a ratio of 10^3^ can be achieved at room temperature. The reversible non-volatile redox reaction between metal and metal oxide may provide additional degrees of freedom for post-fabrication control of properties of solid-state materials. This type of three-terminal device has potential applications in neuromorphic computing and multilevel data storage, as well as applications that require controlling a relatively large current.

## Introduction

Neuromorphic electronics based on artificial synapses can enable non-von-Neumann computing architectures that are highly-parallelized and energy efficient^1–3^. In such systems, the signals transmitted between neurons are represented by currents, the weights of synaptic connections are represented by electric conductance, and the learning function can be achieved by tuning the conductance according to synaptic modification rules^4^. Two-terminal memristive devices can serve as synapses for brain-inspired computing systems^1, 5–7^, but require temporal separation of the learning and signal transmission functions that occur concurrently in natural neural systems^8^. Three terminal resistive switching devices in which a gate controls the channel conductance can more efficiently reproduce synaptic behaviors^9^.

Three terminal resistive switches can also enable multilevel data storage. Multilevel data storage can be achieved with two-terminal resistive memory devices, but the resistance state can be perturbed during the read operation^10^. Since three-terminal resistive switches have separate read and write paths they can offer improved multilevel memory stability. A variety of three terminal resistive switches have been proposed. A three-terminal chemical resistive switch was first proposed in 1960 and termed memistor^11^, but the reliance on a liquid electrolyte limited its applicability. Solid-state memistors were later developed based on WO_3_ thin films^12^, and though high resistance change ratio could be achieved it required operating voltages in the range 10–30 V. Atomic switches have been studied for three-terminal resistive switching devices, but the fabrication is relatively complicated^13^. Ferroelectric field effect transistors are promising for three terminal resistive switching as they provide fast switching speed and high resistance change ratio, but they are usually limited by the retention time^14^. New types of three-terminal switches based on alternative switching mechanisms could lead to enhanced performance and broader application possibilities.

Here we study a three-terminal resistive switch based on solid-state redox reactions between metal and metal oxide. Because of the large resistivity difference between the metal and its oxide, redox reactions can be used to substantially modulate the resistivity of the material. Bauer *et al*. demonstrated that by applying a voltage to a Co/GdOx/Au thin-film stack, oxygen ions in the GdOx layer can be driven towards or away from the Co layer to oxidize or reduce it, and thereby control the magnetic properties of the Co^15^. The evidence of the coexistence of cobalt oxide and cobalt layers, as well as the reversibility of oxidation/reduction of the cobalt film were demonstrated by Bauer *et al*.^15^ and Bi *et al*.^16^. Bauer also proposed and experimentally realized a three-terminal device^17^ that exploited this same phenomenon to induce nonvolatile changes of the resistance of a metal channel. When Co is oxidized, the stable oxides CoO and Co_3_O_4_ are highly resistive comparing to conductive Co metal. However, the changes Bauer observed were relatively modest due to a thick metal back electrode that acted as a current shunt. Here we use the same material system and a three-terminal configuration but with a more suitable layer structure to demonstrate large lateral resistance (*R*_*L*_) modulation using a small gate voltage *V*_*g*_ applied to the top electrode. Recently, a lateral resistance change was observed in gated metal/GdOx/gate structure, but the effect was slow (on the order of minutes) even at elevated temperatures^18^. Here we show that fast resistance switching at room temperature can be obtained, and provide additional insight into the mechanism and design of such devices. By measuring the resistance change rate at different temperatures, the thermally activated nature of the resistance change is observed. We demonstrate reversible resistive switching at room temperature with a ratio of ~10^3^. The resistive switch demonstrated here has potential applications for neuromorphic computing and multilevel data storage and, due to the stability and low resistivity of the metal channel, it also has potential application of controlling a relatively large current.

**Figure 1:**
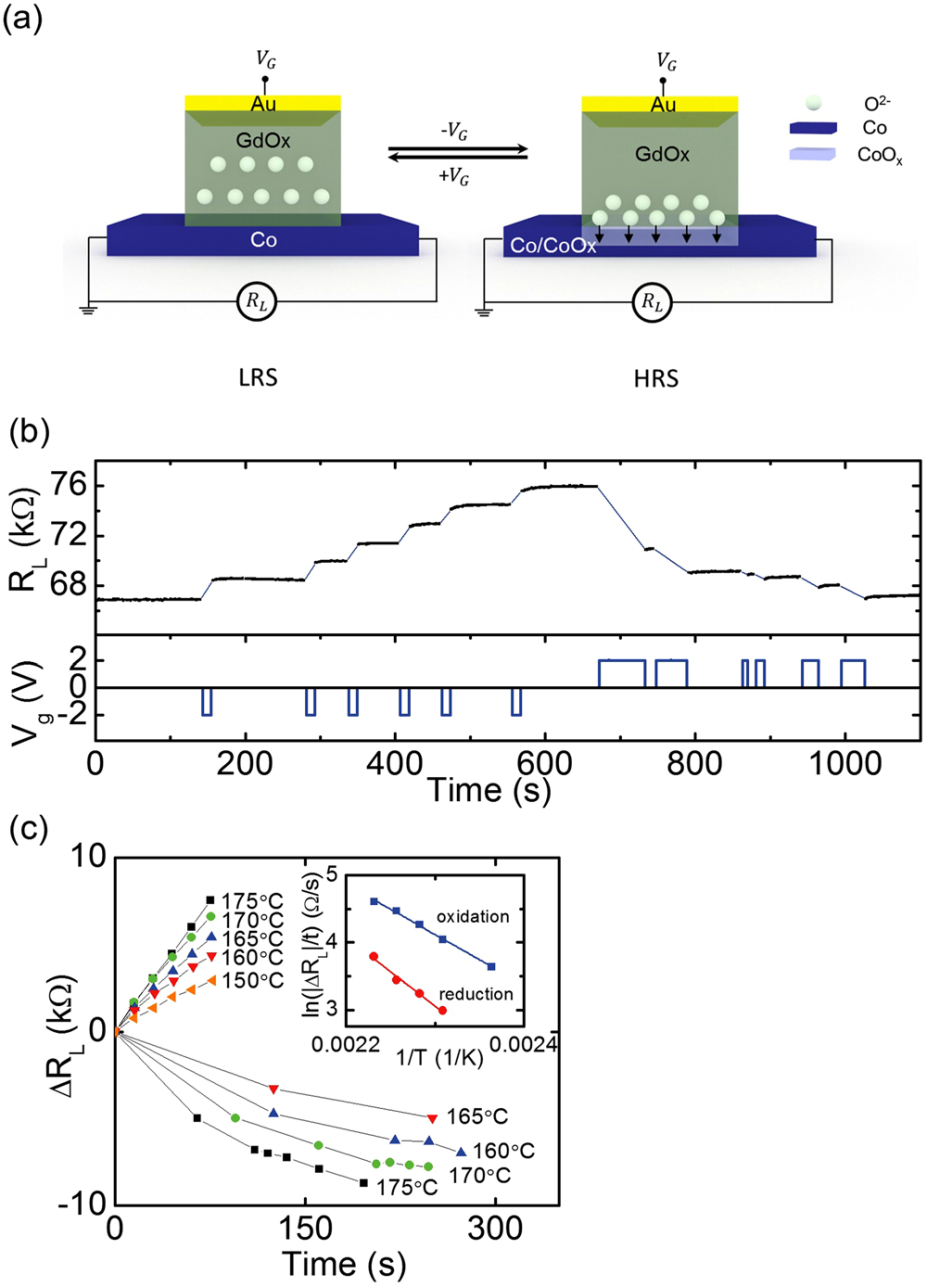
(**a**) Schematic illustration of the operation principle of the three-terminal resistive switch. Oxygen ions in the GdOx layer are shown as white spheres. The application of a negative gate voltage *V*_*g*_ drives the oxygen ions towards the Co layer forming cobalt oxide, and the application of a positive gate voltage reverses the process. (**b**) *R*_*L*_ and *V*_*g*_ as a function of time at 175 °C. (**c**) Resistance change as a function of the duration of −2 V gate voltage (above x-axis) and of +2 V gate voltage (below x-axis). (**c**, Inset) Plot of logarithm of resistance change rate versus inverse temperature under −2 V gate voltage (blue) and under + 2 V gate voltage (red) with linear fittings.

Figure 1(a) illustrates the operation of a resistive switch consisting of a Co channel at the bottom, a GdOx gate oxide that acts as a solid-state ionic conductor^15^, and a Au gate electrode on the top. The channel resistance *R*_*L*_ is measured by probes connected to the two ends of the Co channel. One probe also serves as the ground for the application of gate voltage *V*_*g*_ to the Au electrode on the top. The white spheres inside the GdOx layer indicate schematically the relative locations of the oxygen ions. When *V*_g_ < 0 is applied to the gate electrode, oxygen ions are pumped towards the cobalt channel forming insulating cobalt oxide, which is indicated by the light blue region. The thickness of cobalt metal in the gated region decreases, so the channel resistance *R*_*L*_ increases. When *V*_g_ > 0 is applied, oxygen is driven away from the cobalt layer and the cobalt oxide is reduced. The thickness of cobalt metal recovers and the channel resistance *R*_*L*_ decreases. *V*_g_ can hence control the thickness of the cobalt metal layer and therefore the lateral resistance of the channel.

## Experiments

Two sets of devices were fabricated with different lateral dimensions and gate oxide thickness, labeled as (I) and (II) below. Structure (I) is composed of a 200 μm wide Co(5 nm) wire, Ta(2 nm)/Au(5 nm) pads for making electrical contact, a GdOx(80 nm) layer covering the whole cobalt wire, and a 2.6 mm wide Ta(1.4 nm)/Au(5 nm) top electrode. Structure (II) is composed of a 200 μm wide Co(5 nm) wire, Ta(2 nm)/Au(5 nm) contact pads, a GdOx(10 nm) layer covering the whole cobalt wire, and a 200 μm wide Au(3 nm) top electrode.

We first studied resistance switching of devices (I) at elevated temperatures to enhance the oxygen vacancy mobility^15^. The device was first heated up to 200 °C and after the channel resistance stabilized the device was cooled and maintained at 175 °C. +2 V and −2 V voltage pulses were applied to the top electrode and *R*_*L*_ was monitored between the pulses. When measuring the channel resistance *R*_*L*_, current may flow not only through the Co layer laterally, but also flow through GdOx layer vertically and through the conductive gate. We measured the resistance between the top electrode and the bottom wire to be greater than 10 M Ω both initially and after the application of a voltage pulse, therefore the effect of vertical resistance can be ignored when *R*_*L*_ is much smaller than the gate resistance. Figure 1(b) shows *R*_*L*_ and *V*_*g*_ versus time. *R*_L_ increased (decreased) as negative (positive) *V*_g_ was applied. The direction of resistance change was consistent with the cobalt redox mechanism. The device behaves as a non-volatile multilevel memory because the resistance change Δ*R*_L_ scales with the voltage pulse length, and at *V*_g_ = 0, the resistance is stable. It is possible that Ta in the electrode layer reduces and oxidizes during voltage application. However, because the thickness of Ta is very thin (1.4 nm) compared to the thickness of GdOx(80 nm), it is safe to assume that Ta layer has minimal effect on the oxidation/reduction of Co.

The temperature dependence of the resistance switching of (I) devices was investigated as follows. By applying negative *V*_*g*_, the channel resistance was set to a value *R*_0_ ≈ 68000 Ω. First at 175 °C, *R*_L_ was measured between five −2 V 15 s gate voltage pulses and then the resistance was reduced by applying +2 V pulses to a value slightly below *R*_0_. Repeating these procedures, the resistance increase due to negative *V*_g_ pulses was measured at 175 °C, 170 °C, 165 °C, 160 °C and 150 °C and the resistance decrease due to positive *V*_g_ pulses was measured at 175 °C, 170 °C, 165 °C, and 160 °C. Figure 1(c) shows Δ*R*_L_ as a function of the integrated voltage pulse time. Resistance change rates were then calculated as the average resistance change over time. The inset shows the logarithm of resistance change rate versus inverse temperature when −2 V was applied (blue) and when +2 V was applied (red). The data show a thermally activated Arrhenius-like behavior with an effective activation energy of 0.59 ± 0.02 eV for oxidation and 0.80 ± 0.08 eV for reduction. The reduction is slower and has a higher activation energy than oxidation, which we attribute to the reduction potential of the Co layer, which should act as a built-in potential that biases oxygen ion migration towards the metallic Co layer. Similar results were reported in ref. 18 with a similar device configuration but with a thicker GdOx layer. Our measurements confirm the oxidation and reduction mechanism and serve as a point of comparison for the performance improvement that will be introduced later in following sections of the report.

**Figure 2:**
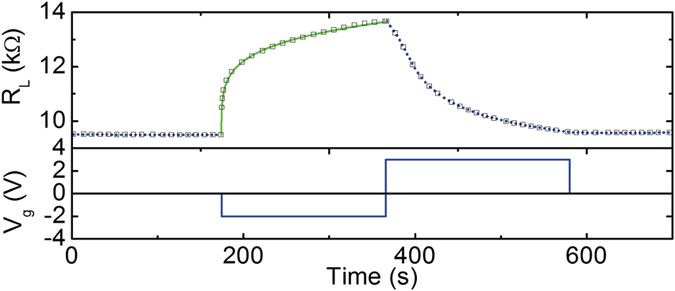
(**a**) *R*_*L*_ and *V*_*g*_ as a function of time at room temperature when −2 V and +3 V pulses are applied in sequence. *R*_*L*_ measurements are shown as black squares. The blue dotted lines are for guiding the eyes. The green line is *R*_*L*_ fitted using logarithm rate law.

In reducing the GdOx thickness from 80 nm to 10 nm, we find the resistance switching behavior of devices (II) can be observed on similar or faster time scales as devices (I) even at room temperature. We attribute this behavior to the larger electric field and higher oxygen vacancy mobility in the thinner GdOx, which was also reported in ref. 15. Figure 2(a) shows the resistance response of a +3 V gate voltage pulse right after a −2 V voltage pulse at room temperature. The resistance increased as *V*_g_ = −2 V gate voltage was applied, and decreased as *V*_g_ = + 3 V gate voltage was applied, and stayed stable after the cycle. The kinetics of oxidation under a negative gate voltage was found to fit well to a logarithm oxidation rate law. The cobalt oxide layer was assumed to be uniform in thickness under the top electrode, and the conduction through the cobalt oxide layer to be negligible compared to the cobalt layer. The total cobalt layer thickness, if all cobalt exist in metallic form, is denoted as *h*_*total*_, and the thickness of cobalt that is oxidized is denoted as *h*_*coOx*_. The resistance of the wire is the sum of two components, which are *R*_*active*_ and *R*_*passive*_. *R*_*active*_ is the resistance of the active region under the top electrode and *R*_*passive*_. is the resistance of the wire outside the gate plus the resistance from electrical contact. Therefore the lateral resistance of the wire ${R}_{L}={R}_{active}+{R}_{passive}$, and ${R}_{active}={\rho }_{Co}\frac{l}{({h}_{total}-{h}_{CoOx})w}$, where *ρ*_*Co*_ is the resistivity of cobalt thin film, *l* is the length of the wire under the gate, and *w* is the width of the wire. The resistivity of Co is assumed to remain constant during the oxidation process because there is no change in the grain boundary density which is the major cause of the increase in the resistivity of thin metal films comparing to bulk materials. The logarithm oxidation rate law is described by:

1 $${h}_{Co{O}_{x}}=K\,\mathrm{ln}(\alpha t+1)+{X}_{0}$$

where ${X}_{0}$ is the initial thickness of cobalt that is oxidized, t is the time during application of the gate voltage, and $K$ and $\alpha$ are rate parameters that depend on the voltage applied and temperature. Here a constant voltage was applied at constant temperature, so $K$ and $\alpha$ are constants. The rate equation gives

2 $${R}_{L}={R}_{passive}+\frac{{\rho }_{Co}l}{w({\rm{C}}-K\,\mathrm{ln}(\alpha t+1))\,}$$

where C is a constant. The green curve in Fig. 2(a) shows the fitting Eq. (2) to the data. A similar logarithm oxidation rate law was observed for metal with a thin oxide layer exposed in air at low temperatures^19–21^. The diminishing rate at longer time indicates that the cobalt oxide formed during the oxidation is probably limiting the switching speed for these devices. This implies that a thinner cobalt layer may lead to faster resistance switching. The positive voltage pulse has a larger amplitude than the negative voltage pulse, but the reduction process takes longer than the oxidation, which is consistent with the observations of (I) devices.

**Figure 3:**
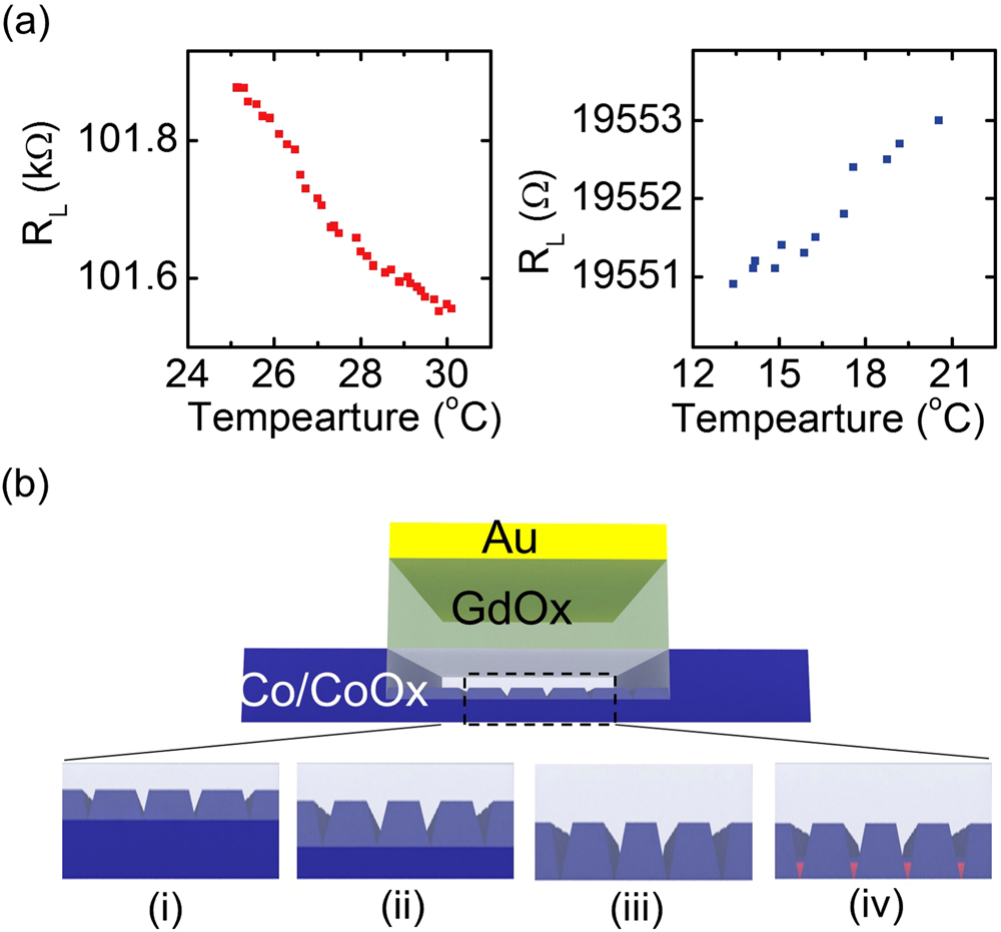
(**a**) R-T curves of the wire at high resistance state (~100 kΩ) and at low resistance state (~19 kΩ) (**b**, i-iii) Schematic illustrations of the possible morphology evolution as the metal is oxidized. (**b**, iv) Schematic illustration of the possible mechanism responsible for the fast resistance decrease after applying the +4 V/−1 V waveform.

Because a reversible high resistance change ratio is desirable for many applications, attempts were made to increase the wire resistance to a very high value and then switch it back. We found that when the *R*_L_ increase is too high (MΩ range), positive *V*_g_ can no longer reduce *R*_L_, which is probably due to loss of electrical connection to the portion of the channel under the gate, which serves as counterelectrode. In order to gain more insight to the phenomena, we measured the temperature-dependence of *R*_L_ as the device goes through oxidation and reduction. First by applying *V*_g_ = −3 V, *R*_L_ was increased to ~100 kΩ. Then by applying a *V*_g_ =+3 V, *R*_L_ was reduced to ~19 kΩ. Figure 3(a) shows resistance versus temperature for both states. At the high resistance state, *R*_L_ decreases as the temperature increases, which indicates semiconducting nature of conduction. At the low resistance state, *R*_L_ increases with increasing temperature, indicating metallic conduction. Similar temperature dependence of resistance at high and low resistance states was also observed in ref. 18 where the voltage was applied at high temperature.

The change in the character of conduction in the Co channel in the high-resistance state, and our observation that after setting the device to even higher *R*_L_ a gate voltage can no longer decrease it, is explained schematically in Fig. 3(b). Initially, the cobalt layer is continuous and metallic with small variation of thickness in the lateral plane, as illustrated in Fig. 3(b) (i). When *V*_g_ < 0 is applied, oxygen ions are driven towards the cobalt layer. The grain boundaries of cobalt are likely to be the fast diffusion paths for oxygen ions and therefore oxidation is likely faster along them. The non-uniformity may get enhanced as the oxidation goes on, as illustrated in Fig. 3(b) (i-ii). When the oxidation time is long enough, islands of metallic cobalt remain while insulating oxide gaps form between the metallic islands as shown in Fig. 3(b) (iii). The conduction of the wire changes from metallic to semiconducting as it is limited by the oxide gaps. As the oxidation goes on, the metallic islands become eventually separated and the electrical connection to the portion of the channel under the gate is lost. As a result, a positive voltage can no longer reduce the wire back to the original state.

**Figure 4:**
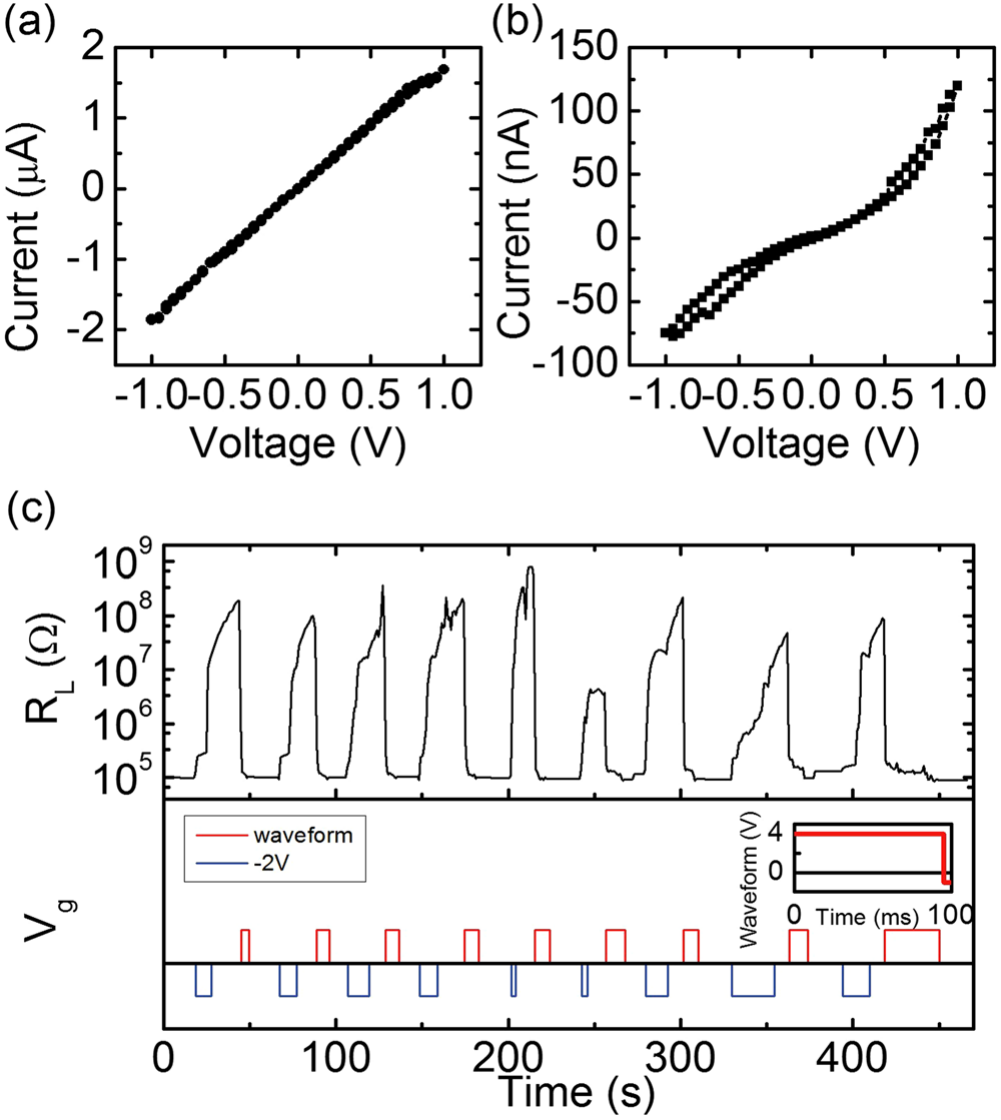
(**a**) I-V curve of the wire at around 500 kΩ. (**b**) I-V curve of the wire at around 20 MΩ. (**c**) Wire resistance change and gate voltage as a function of time demonstrating fast reversible resistance switching at room temperature. (**c**, Inset) +4 V/−1 V waveform used for switching.

Instead of using dc negative and positive voltage to control the wire resistance, we found that by applying negative voltage and an alternating waveform to the top electrode, a reversible resistance change by a factor of 10^3^ can be achieved. Here, a set of devices similar to (II) but with slightly thinner Co (4 nm) was fabricated. By applying a gate voltage of −3 V, the channel resistance can be increased from ~100 kΩ to 10^8^ Ω, but positive dc bias can no longer reduce the resistance. The current-voltage (I-V) characteristics of the Co channel at both a low resistance state and a high resistance state are shown in Fig. 4(a),(b) respectively. At the low resistance state, the I-V curve shows a linear ohmic behavior, while at the high resistance state, a rectifying and hysteretic I-V curve was observed. The shapes of the I-V curves are consistent with the illustrations shown in Fig. 3(b) and the temperature-dependent channel resistance in Fig. 3(a): in the low resistance state, the continuous metallic cobalt layer gives an ohmic I-V behavior, whereas in the high resistance state, and the insulating gaps between metallic islands give raise to a rectifying I-V curve. A similar variation in the I-V behavior in high resistance state and low resistance state was also observed in ref. 18 where the gate voltage was applied at elevated temperatures, and the high resistance state showed threshold switching behavior. The difference in the high resistance state I-V curve could be attributed to the different temperature of these experiments. At room temperature, the diffusion through grain boundaries is probably the dominant path while at elevated temperature bulk diffusivity can be also enhanced and become a feasible way of ionic migration under electric field.

As is known in two-terminal oxide memristive devices, voltage cycling can create conductive filaments or induce charge carrier redistribution across an insulating barrier^22, 23^. We employed a similar strategy to recover electrical contact within the bottom electrode in the high resistance state and to therefore allow recovery of the low-resistance state by gate voltage. Here, instead of applying a dc gate voltage, we applied an alternating +4 V/−1 V waveform to the Au gate electrode, which allowed for successful recovery of the low-resistance state reliably over many cycles (Fig. 4(c)). The waveform has a periodicity of 100 ms, with 95 ms square pulses at +4 V and 5 ms square pulses at −1 V, as shown in Fig. 4(c), inset. Using this protocol, we can reliably toggle the channel resistance by a factor of ~10^3^ times by applying −2 V voltage and the same +4/−1 V waveform, with a switching time less than 30 s. We therefore explain the fast reversible switching as follows. When a negative voltage is applied to the top electrode to oxidized the wire, the electrical connection to the portion of wire under the gate is becoming limited by the insulating gaps between metallic islands as shown in Fig. 3(b) (iii). A positive constant voltage is slow to close the gaps by reduction of the oxide because the process requires formation of electrical connections in the lateral direction. While a −1 V transient pulse may change the defect concentrations inside the gaps and create conductive paths deep in the gaps, which are illustrated as a red layer in Fig. 3(b) (iv). If the negative voltage pulse is followed by a positive voltage pulse, the reduction of oxide can proceed quickly with the help of the conductive paths formed by the negative voltage, and therefore the fast reversible resistance switching with high resistance change ratio can be obtained.

## Discussion

In summary we demonstrated a three-terminal resistive switch based on a metal-redox switch. The device operates by oxidizing a metallic material and reducing the corresponding oxide back to metal with all solid-state operation. The resistance switching was observed to be a thermal activated process. For devices with a thinner GdOx layer, resistance change can be observed at room temperature. By reducing the thickness of the wire layer, switching ratio of ~10^3^ was obtained. Improvements to the device structure may lead to better reliability and faster switching. For example, the Si substrate can be used as the bottom electrode to provide counterelectrode connection even when the active region has high resistivity. In nanoionics devices, switching speed on the order of nanosecond has been demonstrated^24^. Further performance improvement can be expected by reducing the size of the device, by optimizing the thickness of the wire and oxide, by replacing GdOx with better oxygen ion conductor materials, and by optimizing the metal channel material and microstructure. The reversible non-volatile redox reaction between metal and metal oxide provides a way of post-fabrication control of solid-state material composition and properties. This type of three-terminal switch may find applications in neuromorphic computing and multilevel data storage, as well as applications that require a large resistivity change and those involving controlling a relatively large current.

## Methods

### Sample preparation

All the devices were prepared using magnetron sputtering through shadow masks on thermally oxidized Si at room temperature with a background pressure of ~$3\times {10}^{-7}$ Torr. The Ta(2 nm) layer for contact pads was deposited under 2 mTorr Ar. The Co(5 nm) layer, Au(5 nm) layer for contact pads, and top electrode layers were deposited under 3 mTorr Ar. The GdOx layers were deposited by reactive sputtering from a Gd target under 3 mTorr Ar at an oxygen partial pressure of ~$5\times {10}^{-5}$ Torr.

### Electrical Measurement

The Co channel resistance *R*_L_ was measured using a Keithley 2400 sourcemeter by sourcing a 0.05 V voltage. Microprobes were used to make electrical contact, and the contact resistance was measured to be smaller than 20 Ω. *V*_g_ was applied using one of the wire terminals as ground. The top electrode was switched to open loop when the voltage pulses were off (shown as 0 V).

### Data availability statement

The datasets generated during and/or analyzed during the current study are available from the corresponding author on reasonable request.
